# EWS-FLI1 and Activator Protein-1 (AP-1) Reciprocally Regulate Extracellular-Matrix Proteins in Ewing sarcoma Cells

**DOI:** 10.3390/ijms25168595

**Published:** 2024-08-06

**Authors:** Emma E. Croushore, Christopher S. Stipp, David J. Gordon

**Affiliations:** 1 Department of Pediatrics, Division of Pediatric Hematology/Oncology, University of Iowa, Iowa City, IA 52242, USA; emma-croushore@uiowa.edu; 2 Department of Biology, College of Liberal Arts and Sciences, University of Iowa, Iowa City, IA 52242, USA; christopher-stipp@uiowa.edu

**Keywords:** Ewing sarcoma, EWS-FLI1, ribonucleotide reductase, RRM1, activator protein-1 (AP-1), c-Jun, c-Fos

## Abstract

Ribonucleotide reductase (RNR) is the rate-limiting enzyme in the synthesis of deoxyribonucleotides and the target of multiple chemotherapy drugs, including gemcitabine. We previously identified that inhibition of RNR in Ewing sarcoma tumors upregulates the expression levels of multiple members of the activator protein-1 (AP-1) transcription factor family, including c-Jun and c-Fos, and downregulates the expression of c-Myc. However, the broader functions and downstream targets of AP-1, which are highly context- and cell-dependent, are unknown in Ewing sarcoma tumors. Consequently, in this work, we used genetically defined models, transcriptome profiling, and gene-set -enrichment analysis to identify that AP-1 and EWS-FLI1, the driver oncogene in most Ewing sarcoma tumors, reciprocally regulate the expression of multiple extracellular-matrix proteins, including fibronectins, integrins, and collagens. AP-1 expression in Ewing sarcoma cells also drives, concurrent with these perturbations in gene and protein expression, changes in cell morphology and phenotype. We also identified that EWS-FLI1 dysregulates the expression of multiple AP-1 proteins, aligning with previous reports demonstrating genetic and physical interactions between EWS-FLI1 and AP-1. Overall, these results provide novel insights into the distinct, EWS-FLI1-dependent features of Ewing sarcoma tumors and identify a novel, reciprocal regulation of extracellular-matrix components by EWS-FLI1 and AP-1.

## 1. Introduction

Ewing sarcoma is an aggressive bone and soft-tissue cancer that is caused, in the majority of tumors, by an aberrant gene fusion between the *EWSR1* and *FLI1* genes [1]. The EWS-FLI1 oncoprotein functions, in part, as an aberrant transcription factor and is required for tumorigenesis. However, therapeutic targeting of EWS-FLI1, like therapeutic targeting of other transcription factors, has been challenging, and the current therapy for Ewing sarcoma, which has remained unchanged for the past two decades, consists of cytotoxic chemotherapy in combination with surgery and/or radiation [2,3]. Consequently, the identification of novel therapeutic approaches, especially for recurrent and metastatic Ewing sarcoma tumors, is an urgent and unmet clinical need.

Previous work by our group and others identified that Ewing sarcoma tumors are sensitive to drugs that cause DNA replication stress, including inhibitors of ribonucleotide reductase (RNR). RNR, which is composed of RRM1 and RRM2 subunits, catalyzes the rate-limiting step in the de novo synthesis of deoxyribonucleotides and is required for DNA replication and the repair of DNA damage [4,5,6,7,8,9]. However, despite decades of use of RNR inhibitors in the clinic, the polypharmacology and off-target effects of this class of drugs, which include gemcitabine and hydroxyurea, have complicated the identification of the signaling pathways responsible for driving drug sensitivity and resistance [10,11,12,13]. Recently, we used a CRISPR-mediated, gene-knockout-rescue approach to target the RRM1 subunit of RNR in Ewing sarcoma tumors and identified that inhibition of RNR activity upregulates the expression of multiple AP-1 transcription factors, including c-Jun and c-Fos, and inhibits cell growth and colony formation. Furthermore, either the inducible expression of c-Jun and c-Fos in Ewing sarcoma cells or the inhibition of RNR activity is sufficient to downregulate the expression of the oncoprotein c-Myc. However, the effects of AP-1 are pleiotropic, and the critical pathways downstream of AP-1 signaling in the setting of RNR inhibition are unknown, as is the broader role of AP-1 signaling in Ewing sarcoma tumorigenesis.

The AP-1 family of proteins consists of more than twenty members, which belong to the JUN, FOS, ATF, and MAF protein families and function as homo- and hetero-dimeric transcription factors. Notably, in cancer, AP-1 signaling has been implicated in both tumor suppression and oncogenesis in a highly cell- and context-dependent manner [14,15]. AP-1 has many downstream targets and is known to regulate a diverse set of genes related to oncogenesis, apoptosis, and differentiation. The functions of AP-1 in Ewing sarcoma tumorigenesis, beyond the recently described upregulation of AP-1 in the response to DNA replication stress, are largely unknown. However, the EWS-FLI1 oncoprotein has been reported to physically interact with the c-Jun and c-Fos transcription factors. In addition, Tomazou et al. previously described an inverse relationship between EWS-FLI1 expression and the expression of AP-1 target genes, as well as between EWS-FLI1 expression and the expression of AP-1 proteins themselves, and hypothesized that EWS-FLI1 may exert its repressive functions by preventing AP-1 from activating its target genes [16]. Similarly, Wang et al. demonstrated that EWS-FLI1 knockdown significantly upregulates the mRNA expression levels of multiple transcription factors, including several AP-1 proteins [17].

In this study, we used the doxycycline-inducible expression of c-Jun and c-Fos in Ewing sarcoma cell lines, in combination with gene-expression and gene-set-enrichment approaches, to identify genes and pathways that are regulated by AP-1. Notably, we demonstrate that AP-1 and EWS-FLI1 reciprocally regulate the expression of a set of extracellular-matrix proteins. Specifically, we identified that the expression of c-Jun and c-Fos in Ewing sarcoma cells is sufficient to upregulate the expression of multiple proteins, including fibronectin 1 (FN1), vinculin, vimentin, and fibroblast associated protein (FAP), that are downregulated by the EWS-FLI1 oncoprotein. Induced expression of AP-1 also induces phenotypic and morphologic changes in the sarcoma cells while inhibiting cell growth. Furthermore, from a mechanistic standpoint, we identified that the knockdown of EWS-FLI1 broadly dysregulates the expression levels of individual AP-1 proteins. Overall, these results identify downstream signaling pathways induced by DNA replication stress in Ewing sarcoma tumors, connect AP-1 function to the expression of extracellular-matrix components repressed by EWS-FLI1, and link EWS-FLI1 knockdown to the regulation of AP-1 protein levels.

## 2. Results

### 2.1. Genes Upregulated by AP-1 Are Enriched for EMT-Related Genes and Overlap with Genes Repressed by EWS-FLI1

Inhibition of RNR activity in Ewing sarcoma cells causes S-phase cell-cycle arrest, DNA damage, and cell death [14,15,18,19,20,21]. In previous work, using a RRM1 knockout-rescue model and gene-expression analysis, we identified that DNA replication stress upregulates the expression of multiple AP-1 transcription factors in Ewing sarcoma cells and that expression of the canonical AP-1 transcription factors c-Jun and c-Fos is sufficient to downregulate the level of the c-Myc oncoprotein and inhibit cell growth [15]. However, AP-1 transcription factors are well-described regulators of many cellular pathways, and the other downstream targets of AP-1 in Ewing sarcoma cells are unknown [22,23,24]. Consequently, the goal of this current work was to more comprehensively identify the additional pathways regulated by co-expression of c-Jun and c-Fos in Ewing sarcoma cells.

**Figure 1:**
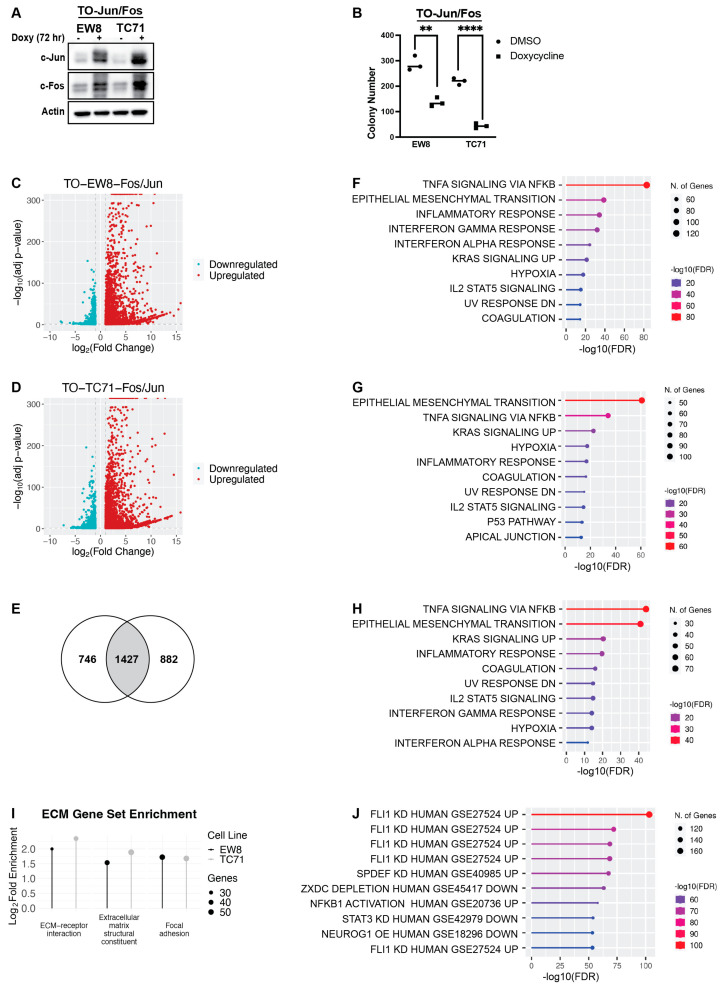
**The set of genes upregulated by AP-1 is enriched for EMT genes and genes repressed by EWS-FLI1.** (**A**) EW8 and TC71 cell lines that express doxycycline-inducible c-Jun and c-Fos (TO-Fos/Jun) were grown in the presence or absence of doxycycline for 72 h. Whole-cell lysates were collected for immunoblotting. (**B**) TO-EW8-Fos/Jun and TO-TC71-Fos/Jun cells were grown in the presence or absence of doxycycline for 10–12 days, after which time colonies were stained with crystal violet and counted using an inverted Olympus CKX41 microscope. ** indicates *p* < 0.001 and **** indicates *p* < 0.0001. (**C**,**D**) Volcano plots of differentially expressed genes (Log_2_(Fold Change) > 1 or <−1, adjusted *p*-value < 0.05) in the TO-EW8-Fos/Jun (**C**) and TO-TC71-Fos/Jun (**D**) cell lines cultured in the presence of doxycycline for 72 h hours compared to cell lines cultured in the presence of the vehicle. (**E**) Venn diagram illustrating the overlap between upregulated genes (Log_2_(Fold Change) > 1, adjusted *p*-value < 0.05) in the TO-EW8-Fos/Jun and TO-TC71-Fos/Jun cells grown in the presence of doxycycline for 72 h compared to cell lines cultured in the presence of the vehicle. (**F**,**G**) Gene sets (HallmarkMSigDB) enriched in the differentially upregulated genes (Log_2_(Fold Change) > 1, adjusted *p*-value < 0.05) in the TO-EW8-Fos/Jun (**F**) and TO-TC71-Fos/Jun (**G**) cell lines grown in the presence of doxycycline for 72 h compared to vehicle. (**H**) Gene sets (HallmarkMSigDB) enriched in the 1427 genes upregulated in both the TO-EW8-Fos/Jun and TO-TC71-Fos/Jun cell lines grown in the presence of doxycycline represented in (**E**). (**I**) Plot demonstrating significant enrichment in ECM-related gene sets among the genes that were significantly upregulated in the TO-EW8-Fos/Jun cells and TO-TC71-Fos/Jun cells grown in the presence of doxycycline for 72 h (adjusted *p*-value < 0.05). Gene sets were derived from KEGG (Extracellular matrix-receptor interaction, Focal adhesion) and GO: Molecular Function (Extracellular matrix structural constituent) analyses. (**J**) Gene sets (TF.Target.TF.Perturbations.Followed.by.Expression) enriched in the 1427 overlapping upregulated genes represented in (**E**).

We used a lentiviral, doxycycline-inducible system to simultaneously overexpress both c-Jun and c-Fos in EW8 and TC71 Ewing sarcoma cell lines (Figure 1A; Table S1). Expression of c-Jun and c-Fos in Ewing sarcoma cells was sufficient, as previously described, to significantly inhibit cell growth in colony-formation assays (Figure 1B) [15]. To identify the genes and pathways regulated by AP-1 in these cell lines (TO-EW8-Fos/Jun and TO-TC71-Fos/Jun), we performed RNA sequencing (RNA-seq) after three days of transgene expression. In both cell lines, the inducible expression of c-Jun and c-Fos resulted in more genes being upregulated than downregulated (Figure 1C,D). There was significant overlap between the differentially expressed genes (DEG) in the TO-EW8-Fos/Jun and TO-TC71-Fos/Jun cell lines (Figure 1E and Figure S1A; Table S2). Gene-set-enrichment analysis (GSEA; Hallmark Database) using the RNA-seq data for the individual TO-EW8-Fos/Jun and TO-TC71-Fos/Jun cell lines lines identified significant (FDR *p* value < 0.05) enrichment for epithelial−mesenchymal transition (EMT), TNF-a signaling, hypoxia, and inflammation-related gene sets (Figure 1F,G). Similar results were obtained from using GSEA to analyze the 1427 overlapping genes that were upregulated in both cell lines (Figure 1H). As shown in Figure 1F–H, each of these analyses identified strong enrichment for the epithelial−mesenchymal transition gene set (HallmarkMSigDB). However, since sarcomas are mesenchymal-derived tumors, we then evaluated the enriched genes from the EMT geneset using KEGG and Gene Ontology (GO): Molecular Function analysis. We identified that these enriched genes from the EMT geneset are further classified as ECM structural constituent, ECM-receptor interaction, and focal adhesion genes, indicating that extracellular-matrix reorganization may be a more specific and accurate description than EMT (Figure 1I).

Next, we analyzed the upregulated genes with transcription-factor-enrichment analysis (TFEA) and identified that the top transcription factor with enrichment for regulation of this set of genes was EWS-FLI1, the driver oncogene in most Ewing sarcoma tumors (Figure 1J) [1,4]. Specifically, the genes that are upregulated by AP-1 are repressed by EWS-FLI1, which supports the hypothesis that the AP-1-driven re-expression of genes repressed by EWS-FLI1 may contribute to RNR-inhibitor toxicity.

**Figure 2:**
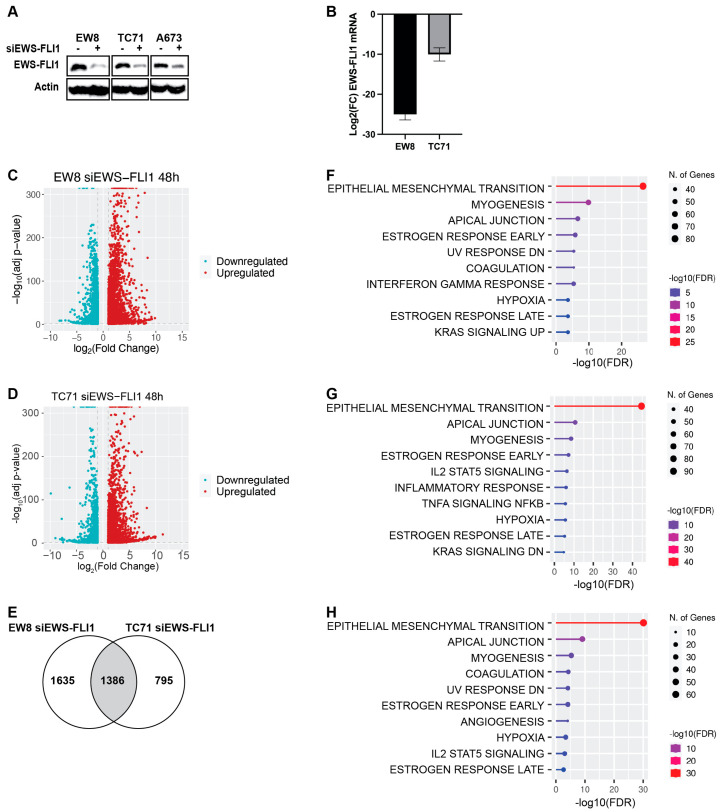
**Genes repressed by EWS-FLI1 are enriched for EMT-related gene sets.** (**A**) EW8, TC71, and A673 cells were treated with siRNA against EWS-FLI1 or a non-targeting control siRNA for 48 h, and then lysates were collected for immunoblotting. (**B**) Log_2_ fold change (FC) in EWS-FLI1 mRNA in EW8 and TC71 cells treated with siRNA against EWS-FLI1 for 48 h. The results are representative of three independent experiments. Error bars represent the mean ± SD of three experimental replicates. (**C**,**D**) Volcano plots of differentially expressed genes (Log_2_(Fold Change) > 1 or <−1, adjusted *p*-value < 0.05) in the EW8 (**C**) and TC71 (**D**) cell lines treated with siRNA against EWS-FLI1 for 48 h compared to cell lines treated with non-targeting control siRNA. (**E**) Venn diagram demonstrating the overlap between genes that were differentially upregulated (Log_2_(Fold Change) > 1, adjusted *p*-value < 0.05) in the EW8 and TC71 cells treated with siRNA against EWS-FLI1 for 48 h compared to genes in the EW8 and TC71 cell lines treated with non-targeting control siRNA. (**F**,**G**) Gene sets (HallmarkMSigDB) enriched in the differentially upregulated genes (Log_2_(Fold Change) > 1, adjusted *p*-value < 0.05) in the EW8 (**C**) and TC71 (**D**) cell lines treated with siRNA against EWS-FLI1 for 48 h compared to cell lines treated with non-targeting control siRNA. (**H**) Gene sets (HallmarkMSigDB) enriched in the 1387 overlap genes that are upregulated in both the EW8 and TC71 cells treated with siRNA against EWS-FLI1 for 48 h compared to those in cell lines treated with non-targeting control siRNA represented in (**E**).

To further investigate the reciprocal regulation of overlapping genes by AP-1 and EWS-FLI1, we performed RNA-seq analysis on EW8 and TC71 parental cell lines that were treated with siRNA targeting EWS-FLI1 or a non-targeting control siRNA (Figure 2A,B). The knockdown of EWS-FLI1 resulted in more genes being upregulated than downregulated (Figure 2C,D), and there was significant overlap between the DEGs for the two cell lines (Figure 2E; Table S3). Notably, GSEA identified that the top gene set upregulated by the knockdown of EWS-FLI1 was the set of epithelial-mesenchymal transition genes (Figure 2F–H), which is the same gene set that was upregulated by expression of c-Jun and c-Fos (Figure 1F–H). Based on the reciprocal regulation of overlapping genes by AP-1 expression and EWS-FLI1 knockdown, we then analyzed whether EWS-FLI1 regulates the expression levels of AP-1 transcription factors. Although the knockdown of EWS-FLI1 dysregulated the expression of multiple AP-1 transcription factors, at the level of both mRNA and protein, the effects on individual transcription factors were not consistent between cell lines in either magnitude or direction (Figure S2A,B, Table 1). A notable exception was that Fosl2, which was recently identified as a suppressor of Ewing sarcoma cell growth, was upregulated by EWS-FLI1 knockdown in both cell lines [25]. Finally, subcellular fractionation experiments did not identify any effect of EWS-FLI1 on the localization of AP-1 proteins (Figure S2C).

### 2.2. The Set of Genes Upregulated by RRM1 Loss, AP-1 Overexpression, and EWS-FLI1 Knockdown Is Enriched for EMT-Associated Genes

**Figure 3:**
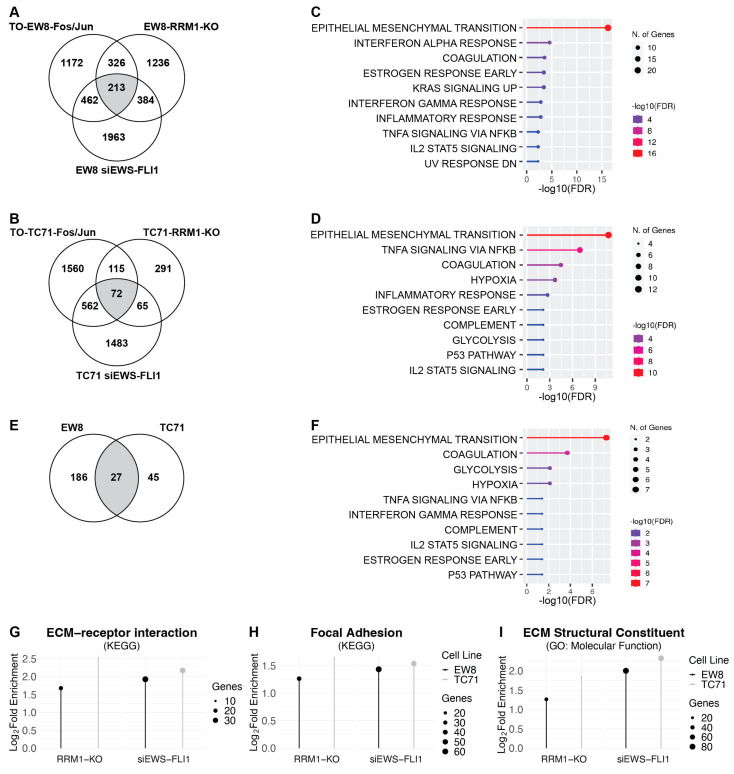
**The list of genes upregulated by RRM1 loss, AP-1 overexpression, and EWS-FLI1 knockdown is enriched for EMT genes.** (**A**) Venn diagram demonstrating the 213-gene overlap between differentially expressed genes (Log_2_(Fold Change) > 1, adjusted *p*-value < 0.05) that were upregulated in the TO-EW8-Fos/Jun cells grown in doxycycline for 72 h, the EW8 cells treated with siRNA against EWS-FLI1 for 48 h, and the EW8-RRM1-KO cell line grown in the absence of doxycycline for 48 h. (**B**) Venn diagram demonstrating the 72-gene overlap between, differentially expressed genes (Log_2_(Fold Change) > 1, adjusted *p*-value < 0.05) that were upregulated in the TO-TC71-Fos/Jun cells grown in doxycycline for 72 h, the TC71 cells treated with siRNA against EWS-FLI1 for 48 h, and the TC71-RRM1-KO cells grown in the absence of doxycycline for 48 h. (**C**) Gene sets (HallmarksMSigDB) enriched in the 213-gene overlap represented in (**A**). (**D**) Gene sets (HallmarksMSigDB) enriched in the 72-gene overlap represented in (**B**). (**E**) Venn diagram demonstrating the 27-gene overlap between (**A**,**B**). (**F**) Gene sets (HallmarkMSigDB) enriched in the 27 overlapping genes represented in (**E**). (**G**–**I**) Plots demonstrating significant enrichment of ECM-related gene sets among the genes that were significantly upregulated in the TO-EW8-Fos/Jun and TO-TC71-Fos/Jun cells grown in doxycycline for 72 h, the EW8 and TC71 cells treated with siRNA against EWS-FLI1 for 48 h, and the TC71-RRM1-KO and EW8-RRM1-KO cell lines grown in the absence of doxycycline for 48 h (ShinyGO 0.77, adjusted *p*-value < 0.05).

To further investigate the similarities in gene-expression patterns between datasets, we overlapped the genes that were upregulated by the expression of AP-1 with those that were upregulated by the knockdown of EWS-FLI1 and those that were upregulated by the knockdown of RRM1 (described in a previous publication) (Table S4) [15]. Notably, overlapping these lists of genes resulted in the identification of 213 (EW8) and 72 (TC71) DEGs that were upregulated under all three conditions (Figure 3A,B). Figure 3C,D demonstrate that this list of overlapping genes was significantly enriched for epithelial−mesenchymal transition genes. Furthermore, overlapping the 213 (EW8) and 72 (TC71) DEG resulted in the identification of a total of 27 genes in common between the three datasets, and this set of genes was also significantly enriched for epithelial−mesenchymal transition genes (Figure 3E,F). Again, further analysis of these genes using Kegg and GO: Molecular Function analysis showed strong enrichment for genes associated with ECM-receptor interactions, focal adhesion, and ECM structural constituents (Figure 3G–I).

**Figure 4:**
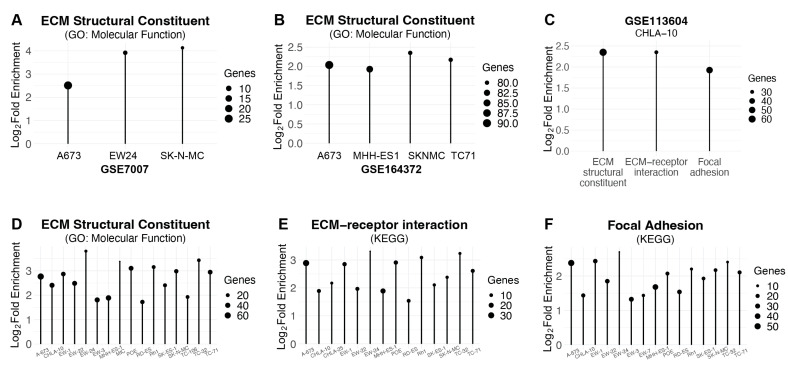
**Genes repressed by EWS-FLI1 are enriched for ECM components in publicly available EWS-FLI1-knockdown data sets.** (**A**) Plot demonstrating significant enrichment in ECM structural components among upregulated genes across three Ewing sarcoma cell lines (A673, EW24, and SK-N-MC) treated with shRNA or siRNA against EWS-FLI1 (ShinyGO 0.77, GSE7007, adjusted *p*-value < 0.05). (**B**) Plot demonstrating significant enrichment in ECM structural components among upregulated genes across four Ewing sarcoma cell lines (A673, MHH-ES1, SKNMC, and TC71) treated with shRNA or siRNA against EWS-FLI1 (ShinyGO 0.77, GSE164372, adjusted *p*-value < 0.05). (**C**) Plot demonstrating significant enrichment in ECM-related gene sets (ECM structural constituent, ECM-receptor interaction, focal adhesion) among significantly upregulated genes in CHLA-10 cells treated with shRNA or siRNA against EWS-FLI1 (ShinyGO 0.77, GSE113604, adjusted *p*-value < 0.05). (**D**–**F**) Plots demonstrating significant enrichment in ECM-related gene sets among significantly upregulated genes across a panel of Ewing sarcoma cell lines treated with shRNA or siRNA against EWS-FLI1 (ShinyGO 0.77, GSE176190, adjusted *p*-value < 0.05).

Next, we evaluated the effect of EWS-FLI1 knockdown on the expression of ECM genes in additional Ewing sarcoma cell lines using multiple, publicly available RNA-seq datasets (Table S5). Analysis of three Ewing sarcoma cell lines, A673, EW24, and SKNMC, treated with siEWS-FLI1 or siControl identified significant enrichment for the ECM structural constituent gene set (GO: Molecular Function) (Figure 4A) [26]. Similarly, additional datasets that treated A673, MHH-ES1, SKNMC, and TC71 cells with shRNA targeting EWS-FLI1 demonstrated enrichment for the ECM structural constituent gene set (GO: Molecular Function) (Figure 4B) [27]. Knockdown of EWS-FLI1 in the CHLA-10 cell line also identified enrichment for multiple ECM gene sets, including the ECM structural constituent, ECM-receptor interaction, and focal adhesion gene sets (Figure 4C) [28]. Finally, analysis of inducible shEWS-FLI1 knockdown in a large panel of Ewing sarcoma cell lines identified enrichment for the same gene sets: ECM structural constituent, ECM-receptor interaction, and focal adhesion (Figure 4D–F) [29]. Overall, analysis of these multiple publicly available datasets, which are based on different Ewing sarcoma cell lines, experimental designs, and knockdown approaches, support the findings from our own experiments that the knockdown of EWS-FLI1 upregulates the expression of genes related to the ECM. In addition, we also evaluated the effect of EWS-FLI1 knockdown on the expression levels of AP-1 transcription factors and identified, as described above for our results with the EW8 and TC71 cell lines, changes that were broad but inconsistent in both direction and magnitude across cell lines and experiments (Figure S3A–D, Table S5).

**Figure 5:**
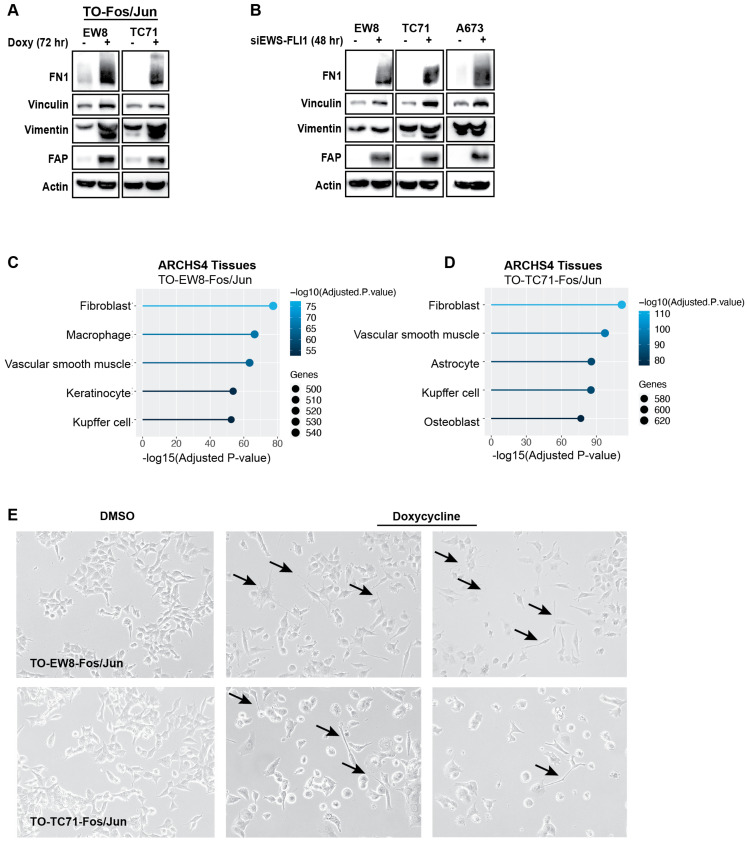
**AP-1 overexpression and EWS-FLI1 knockdown upregulate protein expression of ECM components.** (**A**) TO-EW8-Fos/Jun and TO-TC71-Fos/Jun cells were grown for 72 h in the presence or absence of doxycycline, and then cellular lysates were collected for immunoblotting. (**B**) EW8, TC71, and A673 cells were treated with siRNA against EWS-FLI1 for 48 h, after which point cellular lysates were collected for immunoblotting. (**C**,**D**) Plots showing tissue types enriched in TO-EW8-Fos/Jun (**C**) and TO-TC71-Fos/Jun (**D**) cells grown in doxycycline for 72 h compared to vehicle-treated controls (Enrichr, ARCHS^4^ Tissues). (**E**) TO-EW8-Fos/Jun and TO-TC71-Fos/Jun cells were grown for 72 h in the presence or absence of doxycycline. Black arrows denote elongated cells with an atypical morphology.

Next, we performed immunoblotting to validate the upregulation of ECM genes identified in our RNA-seq analyses. Figure 5A shows that the expression c-Jun and c-Fos in Ewing sarcoma cells is sufficient to upregulate the levels of multiple ECM proteins, including fibronectin, vinculin, fibroblast activation protein, and vimentin. A similar increase in the expression of these ECM proteins was also observed after siRNA-mediated knockdown of EWS-FLI1 in the EW8 and TC71 cell lines (Figure 5B). Consistent with the induction of a more fibroblast-like or differentiated cell state, analysis of the gene-expression data using All RNA-seq and ChIP-seq sample and signature search (ARCHS^4^) revealed that expression of c-Jun and c-Fos in both EW8 and TC71 cell lines generates expression signatures that are most consistent with a fibroblast cell type (Figure 5C,D). In addition, congruent with these gene- and protein-expression changes, we also observed that expressing c-Jun and c-Fos in Ewing sarcoma cells caused distinct and reproducible morphological changes in the cells, in addition to reduced cell growth in colony-formation assays (Figure 1B and Figure 5E).

## 3. Discussion

Ewing sarcoma tumors are sensitive to pharmacologic and genetic inhibition of ribonucleotide reductase (RNR) activity [14,15,19,20,21]. Although small-molecule drugs targeting RNR are used in the clinic, the identification of the biological pathways mediating the effects of RNR inhibitors has been complicated by polypharmacology and the off-target effects of drugs [30,31,32]. However, we recently identified that both the DNA damage, cell-cycle arrest, and cell death caused by RNR inhibitor and the knockdown of either RNR subunit activate AP-1 signaling in a Slfn11-dependent manner [15]. Furthermore, we demonstrated that upregulation of AP-1, even in the absence of RNR inhibition, is sufficient to impair the growth of Ewing sarcoma cells and suppress the expression of oncogenic c-Myc [15,33,34,35,36].

**Figure 6:**
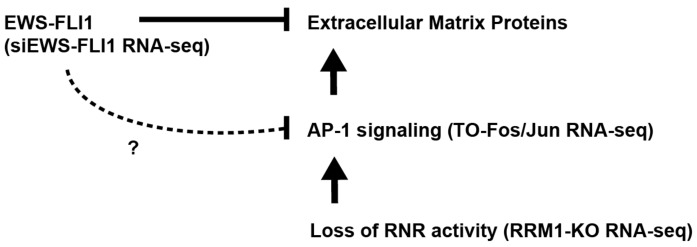
Working model demonstrating the convergence of genes upregulated by EWS-FLI1 knockdown, inhibition or loss of RNR activity, and AP-1 signaling on extracellular-matrix-related signaling pathways in Ewing sarcoma cells. Inhibition or loss of RNR activity in Ewing sarcoma cells results in upregulation of AP-1 protein expression and activity, which subsequently upregulates expression of extracellular-matrix and fibroblast-associated genes, including FN1, vinculin, vimentin, and FAP. These ECM-related genes significantly overlap with EWS-FLI1-repressed genes, demonstrating a form of reciprocal regulation of target genes between AP-1 and EWS-FLI1. Additionally, knockdown of EWS-FLI1 results in the differential expression of various AP-1 proteins at the mRNA and protein levels, suggesting that EWS-FLI1 plays a regulatory role in AP-1 signaling. However, whether this is mechanism is direct or indirect remains to be determined.

In the current study, we used transcriptome- and gene-set-enrichment analysis to comprehensively evaluate the effects of AP-1 on gene expression in Ewing sarcoma cells (Figure 6). Notably, we identified that AP-1 signaling upregulates the expression of extracellular matrix (ECM) components, inhibits cell growth, and induces an elongated, spindle-like morphology in Ewing sarcoma cells. Furthermore, transcriptome and gene set enrichment analysis revealed that a significant number of genes upregulated by AP-1 are known to be repressed by EWS-FLI1. Subsequently, using siRNA-mediated knockdown, we showed that loss of EWS-FLI1 in Ewing sarcoma cells significantly dysregulates the expression of multiple AP-1 transcription factors and induces overlapping, but not identical, transcriptome changes to AP-1 overexpression, including upregulation of ECM proteins.

The AP-1 transcription factor complex comprises the JUN, FOS, ATF, and MAF protein families of bZIP transcription factors, which bind DNA as dimers to activate target genes in response to a range of cellular signals [23,37]. Given the vast array of dimerization partnerships and expression patterns, AP-1 regulates numerous signaling pathways involved in morphogenesis, differentiation, apoptosis, and the cell cycle [22,23,24,37,38]. In malignant cells, AP-1 has been shown to regulate genes related to the cytoskeleton, proliferation, motility, and angiogenesis, although the specific influence on tumor survival appears context- and cancer-dependent [24,39]. We identified that AP-1 inhibits the growth of Ewing sarcoma cells, induces morphologic changes in the cells, and upregulates the expression of genes that are repressed by EWS-FLI1. These findings align with previous reports demonstrating that the knockdown of EWS-FLI1 dysregulates the expression of AP-1 transcription factors, although we identified novel co-regulatory relationships between extracellular matrix genes and both AP-1 and EWS-FLI1 [16,40]. Furthermore, our findings suggest that increased AP-1 signaling may impair tumor progression by upregulating the expression of genes that direct cells into a more differentiated, fibroblast-like phenotype.

Our results align with multiple reports identifying AP-1 as critical to differentiation and maintenance of fibroblasts [41,42,43]. Recent work in the field of human somatic cell reprogramming identified AP-1 as a key driver of fibroblast-specific chromatin accessibility and gene expression via c-Jun occupation of fibroblast-specific enhancers [41]. The same study found that reprogramming of fibroblasts into induced pluripotent stem cells (iPSCs) could be accomplished only by expressing a dominant-negative AP-1 construct alongside necessary pluripotency genes [41,44]. Furthermore, they reported that elevated c-Jun expression occurred in tandem with the expression of EMT-related genes, as we reported here. Mechanistically, AP-1 may also direct fibroblast differentiation through direct transcriptional upregulation of fibroblast-specific genes such as FAP, whose promoter and enhancers AP-1 has been shown to bind [41,45]. Beyond fibroblasts, AP-1 signaling reportedly enhances differentiation of numerous other cell types, including osteoclasts, osteoblasts, hepatocytes, Th2 cells, oligodendrocytes, and keratinocytes [46,47,48,49,50,51,52,53,54,55,56,57,58,59]. With regard to research on differentiation-inducing therapy, upregulation of c-Jun and c-Fos has also been linked to TPA-induced terminal differentiation of myeloid leukemia cells into a macrophage phenotype via PKC signaling [60,61]. The effect of AP-1 on the differentiation of Ewing sarcoma cells will be an area of future investigation.

However, we note that the upregulation of AP-1 signaling could also enhance the invasive or migratory features of Ewing sarcoma tumors, given the similar transcriptome changes between AP-1 signaling and the EWS-FLI1^low^ cellular state described by multiple groups [62]. Ewing sarcoma cells are phenotypically plastic and can switch between functionally distinct cell states that are dependent on the expression level and transcriptional activity of EWS-FLI1. Specifically, EWS-FLI1^high^ cells are proliferative and EWS-FLI1^low^ cells are migratory and invasive, and express ECM proteins [62,63,64,65]. However, although both EWS-FLI1 and AP-1 regulate the expression of ECM genes, only one third of the genes are regulated by both transcription factors, suggesting that significant differences may exist between the effects of EWS-FLI1 and AP-1 on the ECM and cellular phenotype (Figure S4). Future work will dissect these differences between EWS-FLI1 and AP-1 signaling, both in vitro and in vivo using tumor xenograft models.

In summary, we used doxycycline-inducible expression of c-Jun and c-Fos to identify critical genes, proteins, and pathways regulated by AP-1 signaling in Ewing sarcoma cells. Analysis of differential gene expression and gene-set enrichment identified significant overlap between extracellular matrix genes, including fibronectin, vinculin, and vimentin, which are upregulated by AP-1 and repressed by EWS-FLI1. We also identified that AP-1 signaling inhibits cell growth and induces an elongated, spindle-like morphology, suggestive of differentiation, in Ewing sarcoma cells. Furthermore, we identified that knockdown of EWS-FLI1 in Ewing sarcoma cells dysregulates the expression of multiple AP-1 transcription factors. Overall, our work provides novel insights into the role of AP-1 signaling, which is activated by inhibition of RNR, in Ewing sarcoma tumors and establishes a novel link between EWS-FLI1, AP-1, and the regulation of extracellular-matrix proteins.

## 4. Materials and Methods

### 4.1. Cell Lines and Culture

Cell lines were maintained at 37 °C in a 5% CO_2_ atmosphere. The EW8, TC71, and A673 cell lines were provided by Dr. Kimberly Stegmaier (Dana-Farber Cancer Institute, Boston, MA, USA). The cells were grown in DMEM supplemented with 10% FBS, 100 IU/mL penicillin, and 100 μg/mL streptomycin. Cell lines were used within 8–10 passages after thawing. DNA fingerprinting was used to authenticate cell lines. All cell lines were Mycoplasma-negative and were tested every 6 months for contamination (Universal Mycoplasma Detection Kit, ATCC 30-1012K).

### 4.2. Protein Isolation and Immunoblotting

Adherent cells were collected for immunoblotting from cell-culture plates by trypsinization. Total cell number per sample was determined using a Countess II FL automated cell counter to normalize protein loading for immunoblotting. Cells were then pelleted through a 10 min centrifugation at 600 r.c.f. at 4 °C. Supernatants were discarded, then the pellets were resuspended in 1X D-PBS (ThermoFisher Scientific, Waltham, MA, USA) and spun for an additional 10 min at 600 r.c.f. at 4 °C, after which point the supernatants were removed and discarded. The pellets were resuspended in RIPA buffer (Boston BioProducts, Milford, MA, USA) plus protease and phosphatase inhibitors (Halt Protease & Phosphatase Inhibitor Cocktail, EDTA-free; ThermoFisher Scientific) for 20 min at a 1:50 ratio of Halt:RIPA buffer. The samples were centrifuged for 15 min at 17,000 r.c.f. at 4 °C, and the supernatants were collected and stored at −80 °C if this step was not immediately followed by immunoblotting. The samples were loaded onto an SDS-PAGE gel to separate proteins. Following SDS-PAGE separation, gel contents were transferred to polyvinylidene difluoride membranes (Millipore, Darmstadt, Germany). Soluble nuclear, chromatin-bound, and cytoplasmic fractionation was performed using the Subcellular Protein Fractionation Kit for Cultured Cells (ThermoFisher Scientific) according to manufacturer’s instructions. Antibodies to the following proteins were used in the immunoblots: c-Jun (60A8, Cell Signaling Technology, #9165, 1:1000; RRID:AB_2130165), FLI1 (Abcam, #ab133485, 1:1000, RRID:AB_2722650); c-Fos (Santa Cruz #sc-271243, 1:100; RRID:AB_10610067), FOSL2/Fra2 (Cell Signaling Technology, #19967, 1:1000; RRID:AB_2722526), Lamin A/C (Developmental Studies Hybridoma Bank, #MANLAC1, 1:100; RRID:AB_2618203), actin (Cell Signaling Technology, #4970, 1:5000; RRID:AB_2223172), vinculin (Proteintech, 26520-1-AP, 1:5000; RRID:AB_2868558), alpha tubulin (Proteintech Cat #66031-1-Ig, 1:5000, RRID:AB_11042766), vimentin (Developmental Studies Hybridoma Bank, #AMF-17b, 1:1000; RRID:AB_528505), FN1 (Cell Signaling Technology, #26836 (also 26836BF), 1:1000, RRID:AB_2924220), Histone H3 (Cell Signaling Technology, #4499 (also 4499S, 4499P, 4499L), 1:1000, RRID:AB_10544537), and FAP (E1V9V, Cell Signaling Technology, #66562, 1:1000, RRID:AB_2904193). Immunoblots were analyzed and quantified using Fiji (RRID:SCR_002285) (Table S6).

### 4.3. RNA Sequencing and Analysis

The RNeasy Plus Mini Kit (Qiagen, Venlo, The Netherlands) was used to isolate RNA from cell lines. Samples were submitted to the Iowa Institute of Human Genetics Core Facility for sequencing. Samples were barcoded, pooled, and sequenced on an Illumina NovaSeq 6000 (Illumina; San Diego, CA, USA) to obtain a minimum of 30 million, paired-end, 100 bp reads per sample. FastQC was used to assess the quality of the sequencing reads. Reads were then mapped against the human reference genome (hg38) using the STAR aligner (STAR, RRID:SCR_004463) and summarized at the exon level using featureCounts (RRID:SCR_012919). Raw counts were transformed using the cpm function in EdgeR for sample clustering and principal components analysis (PCA). No outlier samples were identified or removed from the analysis. The DESeq2 package (DESeq, RRID:SCR_000154) was used for the identification of differentially expressed genes. Data regarding differentially expressed gene were analyzed for gene-set enrichment and transcription-factor enrichment using ShinyGO 0.77 (RRID:SCR_019213), Enrichr (RRID:SCR_001575), and iDEP.96 [66,67,68]. The significance of enrichment was assessed using a hypergeometric test and false-discovery rate (FDR) for multiple testing corrections. The sequencing data are available on the Gene Expression Omnibus under the accessions GSE263503 and GSE263504.

### 4.4. Analysis of Previously Published RNA Sequencing Data

Processing and analysis of previously published RNAseq data were performed to the above specifications, with slight variations depending on factors such as read length. Data were deposited by the original authors in the Gene Expression Omnibus Repository and were retrieved under the accessions GSE113604, GSE164372, and GSE215881 [15,27,28].

### 4.5. Analysis of Publicly Available Microarray Data

Data were log_2_-transformed, and differential gene expression analysis was then performed using limma (RRID:SCR_010943). Data regarding differentially expressed genes were analyzed for gene-set enrichment and transcription-factor enrichment using ShinyGO 0.77 (RRID:SCR_019213) and Enrichr (RRID:SCR_001575) [66,67,68]. Data were deposited by the original authors in the Gene Expression Omnibus Repository and were retrieved under the accessions GSE176190 and GSE7007 [26,29].

### 4.6. Clonogenic Assay

Clonogenic assays were performed as previously described [15]. Cells were plated in six-well plates in triplicate and then continuously treated with doxycycline or vehicle (DMSO) for 10–14 days. Colonies were then stained with crystal violet and counted using an inverted Olympus CKX41 microscope.

### 4.7. siRNA Transfection

Cells (1.5–3 × 105) were plated one day prior to transfection in six-well plates. Cells were transfected with siRNA using Lipofectamine RNAiMax (ThermoFisher Scientific) according to the manufacturer’s instructions. siEWS-FLI (sense, 5′-GCAGAACCCUUCUUAUGACUU-3′, antisense, 5′-UUUCGUCUUGGGAAGAAUACUG-3′) was obtained from IDT, and siControl was obtained from Cell Signaling Technology (#6568).

### 4.8. TO-RRM1-KO Cell Lines

TO-RRM1-KO cell lines were generated as previously described [15]. A codon-optimized RRM1 gene construct obtained as a gene block (IDT) was inserted into the Lenti-X-Tet-One vector (Takara Biology, Kusatsu, Japan) using NEBuilder HiFi DNA Assembly (NEB). After verification by sequencing, the plasmid was used to make lentivirus, as described in previous publications. CRISPR/Cas9-mediated knockout of RRM1 was then performed using a pLV[CRISPR] plasmid (VectorBuilder, Neu-Isenburg, Germany) that coexpresses Cas9, a guide RNA (GTGAGTTGTATTCGGGCTAC) targeting *RRM1*, and a hygromycin-resistance gene. Lentivirus was prepared as described in previous publications, and cells were selected in 400 mg/mL hygromycin starting 48 h after transduction. The knockout cell lines were then single-cell cloned using flow cytometry (Becton Dickinson FACS Aria). Knockout of RRM1 in multiple clones was then validated using immunoblotting.

### 4.9. Doxycycline-Inducible Expression of c-Jun and c-Fos

TO-Fos/Jun cell lines were generated as previously described [15]. The full-length c-Jun and c-Fos cDNAs, separated by a T2A element, were synthesized and inserted into a pLV-TRE lentiviral vector downstream of the TRE3G doxycycline-inducible promoter (VectorBuilder). The pLVX-EF1a-Tet3G vector (Takara Bio) was used to express the Tet-On 3G transactivator protein from the human EF1 alpha promoter. Lentivirus was prepared as described above. Ewing sarcoma cells were sequentially infected and selected with geneticin 500 μg/mL (pLVX-EF1a-Tet3G) and puromycin 1 μg/mL (pLV-TRE3G-c-Jun-T2A-c-Fos).

### 4.10. RT-qPCR

Total RNA was extracted from cells using a RNeasy kit (Qiagen) following the manufacturer’s instructions. Then, 1 μg of total RNA was reverse-transcribed into first-strand cDNA using random hexamer primers and the SuperScript III Reverse Transcriptase (ThermoFisher Scientific). RT-qPCR was performed on the ViiA 7 Real-Time PCR System (Life Technologies, Carlsbad, CA, USA) using SYBR Select Master Mix (ThermoFisher Scientific). Reactions were performed in triplicate, and gene expression was normalized to GAPDH. The PCR primer sequences are included in Table S7.

### 4.11. Statistical Analysis

A two-tailed Student’s t-test was used to calculate *p*-values for the comparison of two groups. Analyses for more than two groups were conducted with a one-way ANOVA followed by Dunnett’s multiple comparisons test. Statistical analyses were conducted using GraphPad Prism 9.

## Figures and Tables

**Table 1:** Differential levels of mRNA expression of AP-1 family members in Ewing sarcoma cell lines after siRNA-mediated knockdown of EWS-FLI1 (FDR *p*-value < 0.05).

	Log_2_Fold Change	
Gene	EW8	TC71
*ATF1*	−0.7	−0.6
*ATF2*	0.2	0.2
*ATF3*	−2.4	−2.6
*ATF4*	−1.4	−1.2
*ATF5*	−1.2	−2.9
*ATF6*	−0.5	−0.9
*BATF2*	2.1	−1.4
*FOS*	−2.2	0.4
*FOSL1*	0.9	1.9
*FOSL2*	0.9	0.8
*JDP2*	1.2	−1.6
*JUN*	−2.2	−0.8
*MAFA*	1.6	2.1
*MAFB*	−0.9	−0.5
*MAFG*	−1.0	−1.3
*MAFK*	−1.4	−0.3

## Data Availability

The data generated in this study are available within the article and its Supplementary Data Files. The original sequencing data presented in the study are openly available on the Gene Expression Omnibus under the accessions GSE263503 and GSE263504. Original sequencing data presented by our group in a previous study are available under the accession GSE215881 [15]. Publicly available sequencing data reanalyzed in the study were deposited by the original authors in the Gene Expression Omnibus Repository and are available under the accessions GSE113604 and GSE164372 [27,28]. Publicly available microarray data reanalyzed in the study were deposited by the original authors in the Gene Expression Omnibus Repository and are available under the accessions GSE176190 and GSE7007 [26,29]. Sample code used for RNA-seq and microarray analysis can be acquired through GitHub (https://github.com/croushore/rnaseq-gordon.git, 1 May 2024; https://github.com/croushore/microarray-analysis.git, 2 May 2024).

## Supplementary Materials

The following supporting information can be downloaded at: https://www.mdpi.com/article/10.3390/ijms25168595/s1.
