# Supplementary material for: EWS-FLI1 and Activator Protein-1 (AP-1) Reciprocally Regulate Extracellular-Matrix Proteins in Ewing sarcoma Cells
Source: Int J Mol Sci. 2024 Aug 6;25(16):8595. doi: 10.3390/ijms25168595 (PMC11354993; doi:10.3390/ijms25168595)
Supplement: Supplementary file 1 [file ijms-25-08595-s001.zip › Supplementary Figures_EC_071624.pdf]

Supplementary Figure S1

A

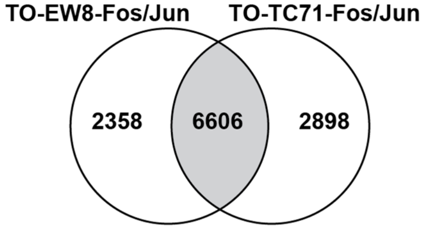

Supplementary Figure S1. Enrichment of overlapping, differentially expressed genes.

(A) Venn diagram demonstrating the 6606-gene overlap in differentially expressed genes (Log_2(Fold Change) > 1 or < -1 (adjusted *P*-value < 0.05) in TO-EW8-Fos/Jun and TO-TC71-Fos/Jun cells treated with doxycycline for 72 hours.

Supplementary Figure S2

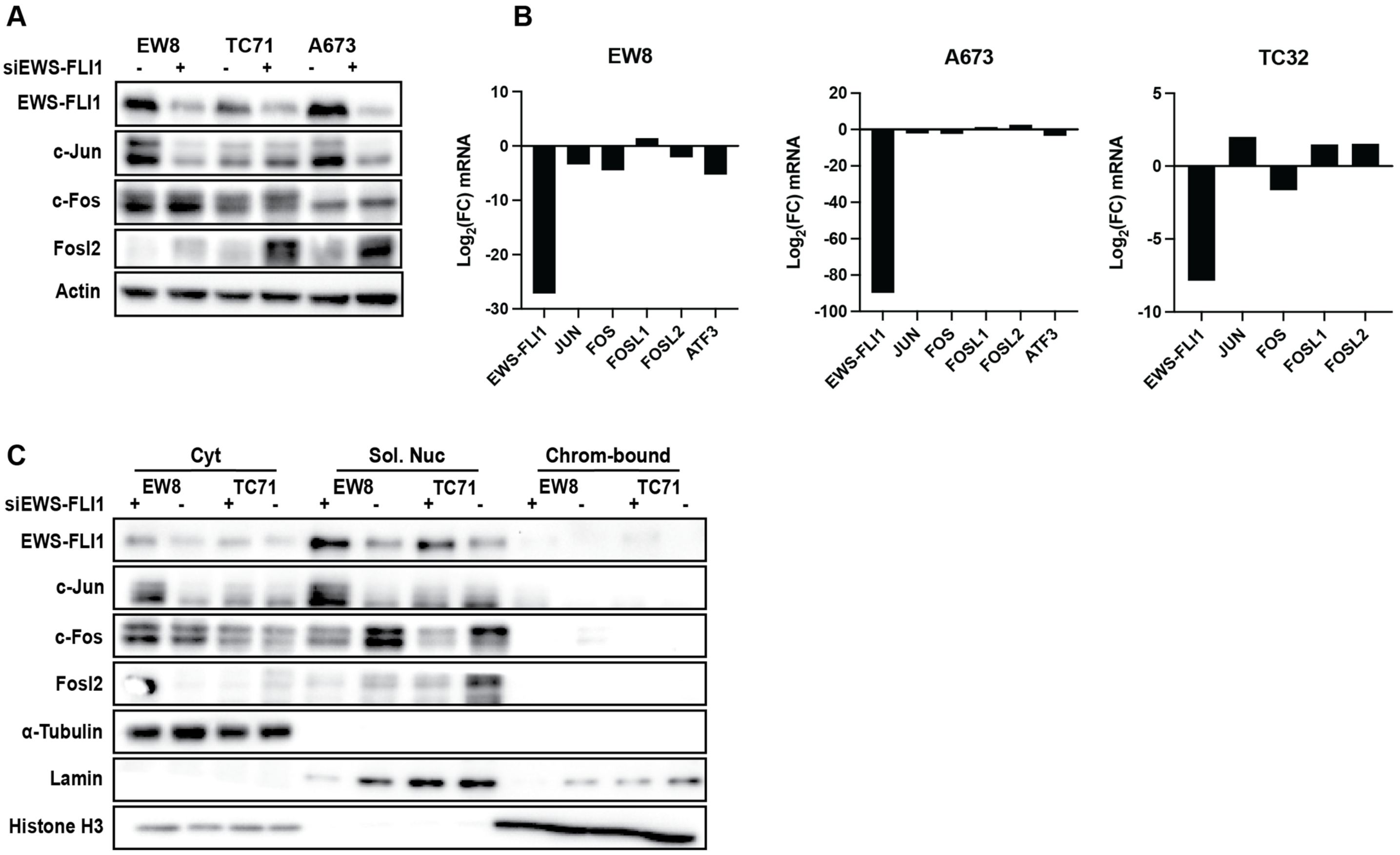

Supplementary Figure S2. Regulation of AP-1 expression by EWS-FLI1.

(A) EW8, TC71, and A673 cells were treated with siRNA against EWS-FLI1 or a non-targeting control for 48 hours. Cellular lysates were then collected for immunoblotting. **(B)** Log_2 fold change (FC) in *EWS-FLI1, JUN, FOS, FOSL1, FOSL2*, and *ATF3* mRNA in EW8, A763, and TC32 cells treated with siRNA against EWS-FLI1 for 48 hours. The results are representative of two independent experiments. Error bars represent the mean ± SD of three technical replicates. **(C)** EW8 and TC71 cells were treated with siRNA against EWS-FLI1 or a non-targeting control for 48 hours. Cellular lysates were then collected and fractionated into subcellular compartments for immunoblotting.

Supplementary Figure S3

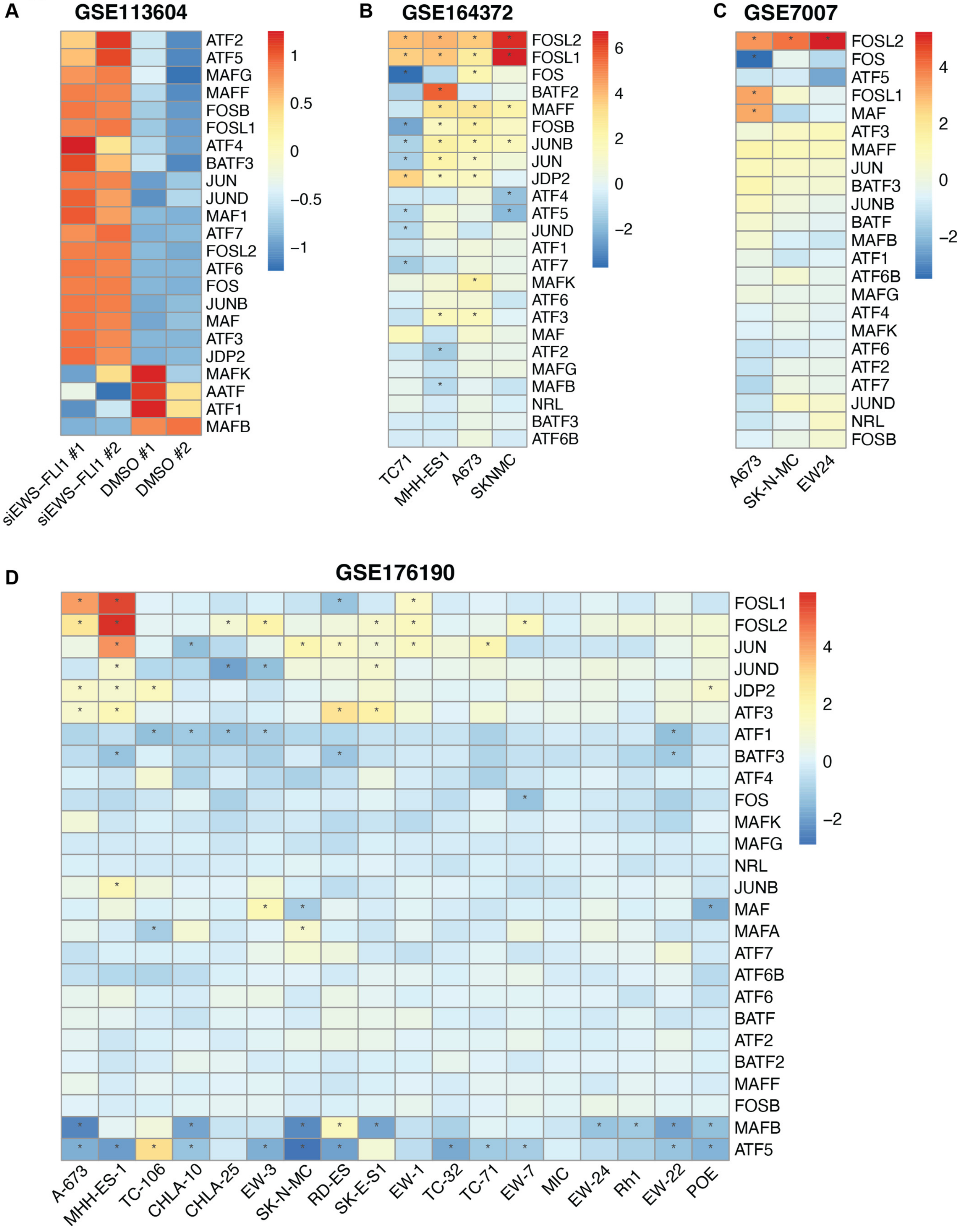

Supplementary Figure S3. EWS-FLI1 knockdown dysregulates mRNA expression of AP-1 transcription factors in published data sets.

(A) Heatmap demonstrating the differential regulation of AP-1 proteins in CHLA-10 cells as a result of EWS-FLI1 knockdown with siRNA. Color scale represents log_2 transformed counts, with each column representing a biological replicate. Gene expression results obtained by reanalysis of RNA-seq data published by Gollavilli et al. (GEO accession number GSE113604)[1]. **(B)** Heatmap representing the differential regulation of AP-1 proteins in TC71, A673, ES1, and SKNMC cell lines as a result of EWS-FLI1 knockdown with siRNA. Color scale represents the range of log_2FC values. Each column represents the log_2FC in mRNA expression for each AP-1 gene in siEWS-FLI1-treated cells relative to controls. Gene expression results obtained by reanalysis of RNA-seq published by Buchou et al. (GEO accession number GSE164372)[2]. **(C)** Heatmap representing the differential regulation of AP-1 proteins in EW24, SKNMC, and A673 cell lines as a result of EWS-FLI1 knockdown with siRNA. Color scale represents the range of log_2FCvalues. Each column represents the log_2FC in mRNA expression for each AP-1 gene in siEWS-FLI1-treated cells relative to controls. Gene expression results obtained by reanalysis of microarray data published by Tirode et al. (GEO accession number GSE7007)[3]. **(D)** Heatmap representing the differential regulation of AP-1 proteins in eighteen Ewing sarcoma cell lines following EWS-FLI1 knockdown (EWS-ERG knockdown in EW-3, CHLA-25, and TC-106 cell lines). Color scale represents the range of log_2FC values. Each column represents the log_2FC in mRNA expression for each AP-1 gene in siEWS-FLI1-treated cells relative to controls. Gene expression results obtained by reanalysis of microarray data published by Orth et al. (GEO accession number GSE176190)[4].

Supplementary Figure S4

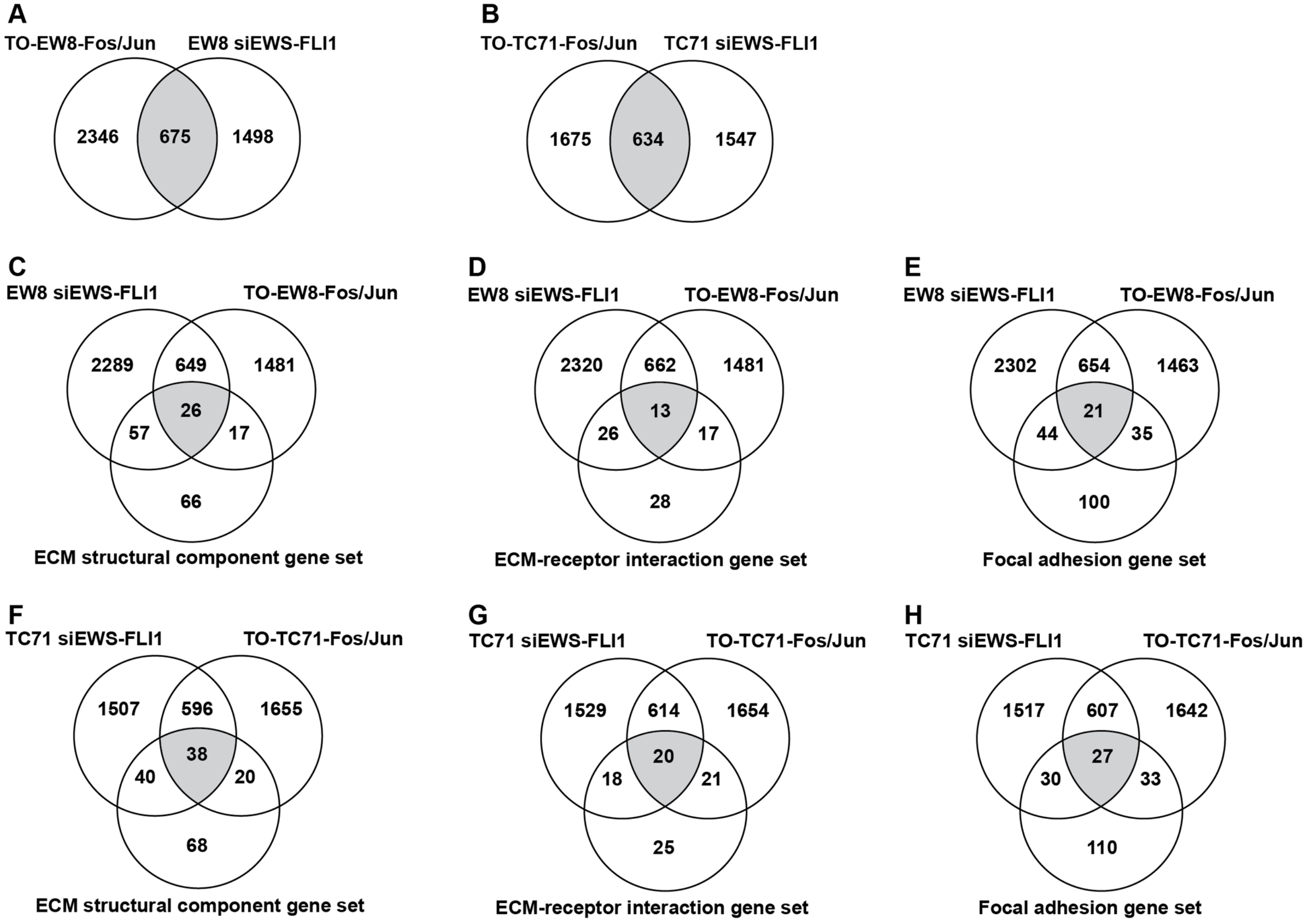

Supplementary Figure S4. Genes upregulated by EWS-FLI1 knockdown and AP-1 upregulation co-regulate some, but not all, genes present in extracellular matrix-related gene sets.

(A, B) Venn diagram demonstrating the overlap in differentially expressed genes (Log_2(Fold Change) > 1 or < -1 (adjusted *P*-value < 0.05) in EW8 siEWS-FLI1 and TO-Fos/Jun cells **(A)** and TC71 siEWS-FLI1 and TO-Fos/Jun cells **(B)**. **(C – E)** Venn diagram demonstrating the overlap in differentially expressed genes (Log_2(Fold Change) > 1 or < -1 (adjusted *P*-value < 0.05) in EW8 siEWS-FLI1 and TO-Fos/Jun cells with ECM-associated gene sets pertaining to ECM structural components **(C)**, ECM-receptor interactions **(D)**, and focal adhesions **(E)**. **(F – H)** Venn diagram demonstrating the overlap in differentially expressed genes (Log_2(Fold Change) > 1 or < -1 (adjusted *P*-value < 0.05) in TC71 siEWS-FLI1 and TO-Fos/Jun cells with ECM-associated gene sets pertaining to ECM structural components **(F)**, ECM-receptor interactions **(G)**, and focal adhesions **(H)**.

References

1. Gollavilli, P.N.; Pawar, A.; Wilder-Romans, K.; Natesan, R.; Engelke, C.G.; Dommeti, V.L.; Krishnamurthy, P.M.; Nallasivam, A.; Apel, I.J.; Xu, T.; et al. EWS/ETS-Driven Ewing Sarcoma Requires BET Bromodomain Proteins. *Cancer Research* **2018**, *78*, 4760–4773, doi:10.1158/0008-5472.CAN-18-0484.
2. Buchou, C.; Laud-Duval, K.; van der Ent, W.; Grossetête, S.; Zaidi, S.; Gentric, G.; Corbé, M.; Müller, K.; Del Nery, E.; Surdez, D.; et al. Upregulation of the Mevalonate Pathway through EWSR1-FLI1/EGR2 Regulatory Axis Confers Ewing Cells Exquisite Sensitivity to Statins. *Cancers (Basel)* **2022**, *14*, 2327, doi:10.3390/cancers14092327.
3. Tirode, F.; Laud-Duval, K.; Prieur, A.; Delorme, B.; Charbord, P.; Delattre, O. Mesenchymal Stem Cell Features of Ewing Tumors. *Cancer Cell* **2007**, *11*, 421–429, doi:10.1016/j.ccr.2007.02.027.
4. Orth, M.F.; Surdez, D.; Faehling, T.; Ehlers, A.C.; Marchetto, A.; Grossetête, S.; Volckmann, R.; Zwijnenburg, D.A.; Gerke, J.S.; Zaidi, S.; et al. Systematic Multi-Omics Cell Line Profiling Uncovers Principles of Ewing Sarcoma Fusion Oncogene-Mediated Gene Regulation. *Cell Reports* **2022**, *41*, 111761, doi:10.1016/j.celrep.2022.111761.
